# Telemedicine Use and the Perceived Risk of COVID-19: Patient Experience

**DOI:** 10.3390/ijerph20043061

**Published:** 2023-02-09

**Authors:** Hassan Hosseinzadeh, Zubair Ahmed Ratan, Kamrun Nahar, Ann Dadich, Abdullah Al-Mamun, Searat Ali, Marzieh Niknami, Iksheta Verma, Joseph Edwards, Mahmmoud Shnaigat, Md Abdul Malak, Md Mustafizur Rahman, Anthony Okely

**Affiliations:** 1 School of Health & Society, University of Wollongong, Wollongong, NSW 2522, Australia; 2 Institute of Child and Mother Health (ICMH), Matuail, Dhaka 1362, Bangladesh; 3 School of Business, Western Sydney University, Penrith, NSW 2751, Australia; 4 School of Business, University of Wollongong, Wollongong, NSW 2522, Australia; 5 The Practice-Bundanoon, Bundanoon, NSW 2578, Australia; 6 Department of Geography and Environment, Jagannath University, 9-10 Chittaranjan Ave, Dhaka 1100, Bangladesh; 7 International Centre for Diarrhoeal Disease Research, Bangladesh (icddr,b), Dhaka 1212, Bangladesh

**Keywords:** telemedicine, COVID-19, risk perception, telecare

## Abstract

Introduction: The COVID-19 outbreak resulted in an increased demand for telemedicine worldwide. Telemedicine is a technology-based virtual platform that allows the exchange of clinical data and images over remote distances. This study aims to examine the impact of the perceived risk of COVID-19 on telemedicine use in Bangladesh. Methods: This explanatory study was conducted in hospital settings across Dhaka city in Bangladesh. Patients were eligible to participate if they were aged 18 years or over and had used telemedicine in a hospital at least once since the COVID-19 outbreak. Outcome variables included sociodemographic, the perceived risk of COVID-19, and telehealth use. Study data were collected using an online and paper-based survey. Results: A total of 550 patients participated in this study, mostly male (66.4%), single (58.2%), and highly educated (74.2%). The means of the different domains of telemedicine use reflected a high degree of perceived benefit, accessibility, and satisfaction but a lower degree of privacy and discomfort, care personnel expertise, and usability. COVID 19 perceived risk predicted between 13.0% and 26.6% of variance in telemedicine domains, while the effects of demographic variables were controlled or removed. The perceived risk of COVID-19 was negatively correlated with privacy and discomfort, as well as care personnel concerns. Low and high levels of perceived COVID-19 risk were less likely to encourage the use of telemedicine as a risk reduction tool. Discussion: The participants were mainly satisfied with telemedicine, finding it beneficial and accessible; however, many were concerned about privacy, care personnel expertise, and its usability. The perceived risk of COVID-19 was a strong predictor (contributor) of telemedicine use, suggesting that risk perception can be used to encourage telemedicine use as a risk reduction strategy during pandemics; however, a medium level of risk was more promising.

## 1. Introduction

The COVID-19 pandemic along with global improvements in technology and access to healthcare triggered a sharp increase in telemedicine. Telemedicine is “the provision of health services, where distance is a critical factor, by all healthcare professionals who use information and communication technologies for the exchange of valid information for the diagnosis, treatment and prevention of diseases” [1]. Telemedicine is a technology-based virtual platform, which allows the exchange of clinical data and images over remote distances [2,3]. It is delivered via various modes, including telephone, smartphone, and videoconferencing [4,5]. There are three types of telemedicine including synchronous, asynchronous, and remote monitoring [2]. Asynchronous is known as “store-and-forward”, which is used for patient intake or follow-up care. Synchronous happens live, where a patient interacts with a doctor. Remote monitoring involves tracking a patient’s health data via a review of test results and images collected remotely or video monitoring of the patient [5]. The advantages of telemedicine are evident by its effectiveness as a therapeutic intervention, being cost-effective, improving compliance, enhancing quality of care, and saving on travel time and the waiting period during physical consultations [6,7,8,9]. Telemedicine has great potential to address health inequalities by reaching disadvantaged groups who often have limited access to a health service due to geographic and affordability barriers [10,11,12]. However, it still faces many challenges, including regulatory issues [13], confidentiality risks, difficulties in assessing clinicians’ telemedicine competencies, compliance management, and difficulties in obtaining patient consent [14,15]. Telemedicine is part of the digital healthcare ecosystem that is evolving worldwide [16]. The focus here is on using telemedicine during COVID-19 and its use beyond the pandemic.

Historically, telemedicine had a slow adoption rate prior to the COVID-19 pandemic with uneven growth, globally [17]. It was used during previous outbreaks such as the severe acute respiratory syndrome (SARS) in 2003 as a transmission risk reduction strategy [18,19]. However, telemedicine was a hasty reaction to COVID-19 worldwide [17]. Globally, the demand for telemedicine surged during the COVID-19 pandemic [20]. For instance, relative to the prepandemic period, telemedicine service use increased by: 38 times in the United States of America; 230% in Argentina [21]; and 122% in Canada [22] during COVID-19 compared to the prepandemic period.

COVID-19 created unprecedented disruption for global health, with approximately 635 million confirmed cases and 6.61 million deaths globally by November 2022 [23]. COVID-19 is highly contagious and transmitted via respiratory droplets generated during coughing and sneezing by symptomatic and asymptomatic patients [24]. To curtail the transmission of the virus, various public health initiatives were introduced, including lockdowns, social distancing, contact tracing, and quarantine [25]. Telemedicine was also used to mitigate increasing infection risks for both patients and healthcare staff [26]. Studies showed that telemedicine reduced COVID-19 infections by preventing travelling to or from hospitals [27,28].

Similarly, Bangladesh, a limited resourced and densely populated (~170 million people) nation faced enormous challenges from the early days of the pandemic [29]. The rate of transmission was very high among community members and healthcare staff. As of 22 November 2022, there had been 2,036,393 confirmed cases of COVID-19 with 29,431 deaths [30]. To ease transmission, telemedicine services were introduced by the Bangladeshi government. Despite the benefits associated with telemedicine, its use is often hindered by concerns about privacy and confidentiality, limited operational capacity, as well as limited digital literacy [31]. There is limited clarity about the role of the perceived risk of COVID-19 on telemedicine. This study aims to addresses this by examining the effect of the perceived risk of COVID-19 on telemedicine use in Bangladesh.

## 2. Method

### 2.1. Design

This exploratory study involved the collection and analysis of quantitative data via a survey, distributed online and via post. This study was reviewed and approved by the Health & Medical Human Research Ethics Committee (Ethics Number: 2021/231) at the University of Wollongong, Australia. It was assessed and approved by the participating hospitals before data collection.

### 2.2. Setting and Sample

The study was conducted in hospitals across Dhaka city in Bangladesh. A power analysis estimated that a sample size of 300 patients would be sufficient to detect a minimum correlation of 15% between COVID-19 perceived risk and telemedicine use, with 85% power at the 5% significance level [32]. A total of 575 patients participated in the study, with 550 completing the survey.

### 2.3. Inclusion Criteria

Hospitals were eligible to be represented in this study if they delivered telemedicine and were in Dhaka city in Bangladesh. Patients were eligible to participate if they were aged 18 years or over, had used telemedicine in a hospital at least once since the COVID-19 outbreak, and did not have a cognitive impairment (e.g., Alzheimer’s disease).

### 2.4. Hospital Recruitment

Hospitals were recruited with the assistance of the Institute of Child and Mother Health Network, which provided a list of eligible hospitals. An invitation letter and an information sheet were sent to eligible hospitals. If a hospital representative did not respond after two weeks of the initial invitation, they were contacted via telephone or email. A researcher visited each senior hospital representative who expressed an interest in this study. During this visit, the hospital representative was briefed about the study and invited to ask questions. Participating hospitals provided list of eligible patients and their contact details.

### 2.5. Patient Recruitment

A researcher contacted all potential participants via telephone to invite their participation. Eligible participants were followed up after two weeks if they did not answer the first telephone call.

### 2.6. Data Collection

Data were collected via an online and a postal survey, pending participant preference. The survey required approximately 20 to 25 min to complete. The online survey was completed via Qualtrics, which is a robust web-based survey creation software that allows creation of research surveys and collection of data [33]. Specifically, participants received a hyperlink via an invitation letter to access the survey and information sheet—consent was implied. Participants who preferred the postal survey received this via mail, accompanied by the information sheet and a prepaid return envelope to post the completed survey—consent was implied.

### 2.7. Outcome Measures

*Demographic Variables*: Patient data included gender, age, employment, education level, family income, health issues when telemedicine was used, and modes of telemedicine used.

### 2.8. Perceived Risk of COVID-19

The COVID-19 own risk appraisal scale (CORAS) was used to measure participants’ perceived risk of COVID-19 [34]. It is comprised of six items that are scored using a five-point Likert scale. A Likert scale is a rating scale which uses a 5- or 7-point scale to measure participants’ opinions. It consists of questions or statements with five or seven answer options. Participants can select the option that describes how they feel about each of the statements or questions [35]. Total CORAS scores were calculated by adding the score of each item, with higher scores indicating a higher perceived risk of COVID-19. This valid scale focuses on one’s own perceived risk, rather than perceived risk to others, which might not necessarily guide behaviour during a pandemic.

### 2.9. Acceptability of Telehealth Use (Patient)

The whole systems demonstrator SUTAQ—service user technology acceptability questionnaire (WSD-SUTAQ) [36] was used to measure the acceptability of telemedicine. This reliable scale has six subscales, including: perceived benefit (5 items); accessibility (4 items); privacy and discomfort (4 items); care personnel concerns (3 items); usability (3 items); and satisfaction (3 items). The SUTAQ questionnaire consists of 22 statements with both negatively and positively worded items. Participants rated their level of agreement with each statement on a six-point Likert scale.

### 2.10. Study Measures Translation

The measures available in English were translated into Bengali. To ensure consistency, two academics who are fluent in both Bengali and English performed the translation. One academic initially translated the measures from English into Bengali; the second academic then translated these measures back into English. After this, both academics compared the English versions of the measures and addressed differences [37]. Study survey and flyer are included in File S1.

### 2.11. Data Analysis

Study data were analysed using IBM SPSS Statistics package version 28. First, univariate statistics including frequencies, mean, standard variation, and range were performed to describe participants’ demographic variables, as well as study variables’ descriptive statistics and distributions. Second, an independent sample *t*-test and a one-way ANOVA using Dunnett post hoc analyses were conducted to assess the association of COVID-19 perceived risk and different domains of telemedicine use with demographic characteristics and type of disease. Third, multiple regression analyses were used to examine whether COVID-19 perceived risk was a reliable and significant predictor for different domains of telemedicine use while controlling for sociodemographic variables. COVID-19 perceived risk and all of the demographic variables (as dummy variables) were entered into the regression equation simultaneously for each of domains of telemedicine use including perceived benefit; accessibility; privacy and discomfort; care personnel concerns; usability; and satisfaction. A dummy variable also known as indicator variable is a numerical variable that is generated to represent a categorical variable in regression analysis. It is a 0 or 1 value indicating the absence or presence of a categorical variable, respectively, in regression analysis. For instance, for gender, female can be defined as 1 and male can be defined as 0 to divide the sample into two subsamples, female and male, and compare them [38]. We were interested in calculating unique predictive power of COVID-19 perceived risk for each domain of telemedicine. As such, we reported squared partial correlations (unique variance), which shows relationship between each predictor and the outcome while controlling for all other predictors in the model, whereas common variance shows relationship between all predictors in the equation and the outcome [39]. T-test analyses were two sided. For regression analyses all variables were entered in a single step. A *p*-value less than 0.05 was considered statistically significant. Multiple linear regression analyses were performed using the following equation. yi = β0 + β1xi1 + β2xi2 + ... + βpxip + ϵ. In the formula, I indicates n observations; yi indicates dependent variable; xi represents explanatory variables; β0 indicates y-intercept; βp represents slope coefficients for each explanatory variable; and ϵ represents the model’s error term (the residuals) [40].

## 3. Results

### 3.1. Demographic Variables

A total of 550 patients participated in this study (see Table 1). The majority of the participants were male (66.4%), aged 18–25 years (46.4%), single (58.2%), unemployed (49.6%), from a high-income family (47.8%), and held a university and/or college qualification (74.2%), while, of the participants who had used telemedicine for various health issues, the most prominent were heart disease (18.9%), skin disease (15.5%), as well as stomach and/or bowel disease (14.0%). The most common mode of telemedicine was a telephone call (55.4%), followed by a live video chat (18.9%).

### 3.2. Perceived Risk of COVID-19

The perceived risk of COVID-19 was moderately high among the participants. This is because the mean COVID-19 perceived risk was 21.5 out of 30, with a standard deviation of 3.6. Most participants disagreed that they would not contract COVID-19 (68.0%; see Table 2). Similarly, 63.1% pictured themselves contracting COVID-19 very easily. However, just over half of the participants (57.3%) perceived their chances of contracting COVID-19 as high and only 37.1% reported being vulnerable to COVID-19. ANOVA analysis showed that only education and income were significantly associated with the perceived risk of COVID-19. Participants with high education and family income levels reported a greater perceived risk of COVID-19, relative to those who were illiterate (had no education) and of low income. As such, the perceived risk of COVID-19 increased with increased education and family income.

### 3.3. Perceptions towards Different Domains of Telemedicine Use

Among the participants, the means of the different domains of telemedicine use reflected a high degree of perceived benefit, accessibility, and satisfaction but a lower degree of privacy and discomfort, care personnel expertise, and usability (see Table 3). In relation to perceived benefit, more than half of the participants moderately or strongly agreed that telemedicine led to more active involvement in their health (53.0%) and better health monitoring (50.2%); consequently, they recommended telemedicine to others (62.4%) and moderately or strongly agreed that it can be a good addition to regular health services (53.0%). Similarly, in terms of accessibility, more than half of the participants moderately or strongly agreed that telemedicine saved time (56.1%), increased access to care (55.8%), improved health (53.8%), and eased access to healthcare professionals (55.4%). Furthermore, regarding satisfaction, over half of the participants: received sufficient information about telemedicine use (58.9%); were satisfied with telemedicine (61.1%); and moderately or strongly agreed that telemedicine can be trusted (61.5%).

Many participants moderately or strongly agreed that telemedicine interfered with their daily routine (34.2%), invaded their privacy (30.5%), made them uncomfortable (25.5%), and spurred confidentiality concerns (33.8%). Similarly, many were moderately or strongly concerned about: their healthcare professional’s expertise in delivering telemedicine (44.0%); their healthcare professional’s knowledge of their medical history (42.5%); and the continuity of their care (42.0%). Within the domain of usability, only 39.1% of the participants moderately or strongly agreed that telemedicine can replace regular healthcare and about one-third moderately or strongly agreed that telemedicine enabled them to be less concerned about their health (32.1%).

### 3.4. Distribution of Telemedicine Domains across Demographic Variables

*t*-test analyses showed that, relative to their female counterparts, male participants were likely to have higher scores regarding accessibility, usability, and satisfaction but lower scores in care personnel concerns (see Table 4). ANOVA analyses using post hoc Dunnett tests showed that married participants were more likely to find telemedicine less accessible but were less concerned about privacy and discomfort. Furthermore, those who were educated and had a high family income were more likely to have higher scores in the domains of perceived benefit, usability, and satisfaction. Accessibility was associated with higher education leading to higher accessibility scores. However, those who were educated and had a high family income were more likely to be concerned about privacy and discomfort. Older participants were more likely to be concerned about their healthcare professional’s expertise in delivering telemedicine.

### 3.5. Comparison of Different Domains of Telemedicine Use across Different Types of Diseases

*t*-test analysis indicated that, compared to other health issues, participants with arthritis were likely to recognise the benefits of telemedicine, while those with pain were unlikely to recognise these benefits (see Table 5). Participants with lung disease were less likely to recognise telemedicine’s accessibility, while those with infectious disease were likely to recognise telemedicine’s accessibility. Participants with arthritis, hyperlipidaemia, immune disease, and pain were less likely to raise privacy and discomfort concerns, while those with mental health issues, sexual health issues, or infectious disease were more likely to raise privacy and discomfort concerns. Only participants with eye disease raised concerns about healthcare professionals’ expertise in delivering telemedicine. Those with heart disease recognised the usability of telemedicine, while those with mental health or sexual health issues perceived less usability. Only participants with arthritis appeared to be more satisfied with telemedicine, relative to those with other health issues.

### 3.6. Predictive Power of COVID-19 Perceived Risk

The perceived risk of COVID-19 strongly and significantly predicted the different domains of telemedicine use (see Table 6). After controlling the effect of demographic variables, the perceived risk predicted between 13.0% and 26.6% of variance in telemedicine use including perceived benefit (19.4%), accessibility (26.6%), privacy and discomfort (16.8%), care personnel concerns (13.0%), usability (15.5%), and satisfaction (19.6%). Furthermore, the perceived risk of COVID-19 was negatively correlated with privacy and discomfort, as well as care personnel concerns. As such, participants with a greater perceived risk of COVID-19 were less worried about privacy and discomfort concerns, or their healthcare professional’s expertise in delivering telemedicine. Finally, the multiple regression analysis found that gender, age, marital status, education level, and family income significantly influenced the predictive power of the perceived risk of COVID-19. Specifically, the perceived risk strongly predicted: perceived benefit among educated participants; privacy and discomfort concerns among older participants (≥41 years), those employed part-time or casually, and those who were married; care personnel concerns among older participants (≥41 years); and satisfaction among those with a middle family income.

**Figure 1:**
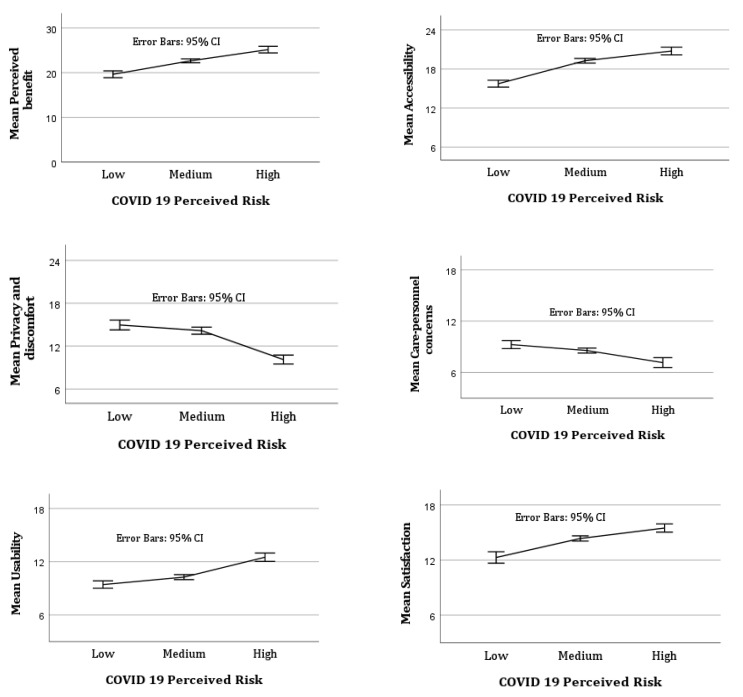
Mean plot of different domains of telemedicine use by COVID-19 perceived risk.

A mean plot showed that the mean of perceived benefit, accessibility, usability, and satisfaction increased exponentially with an increase in the perceived risk of COVID-19 (see Figure 1). However, the mean of privacy and discomfort concerns and concerns about healthcare professionals’ expertise in delivering telemedicine decreased exponentially when the perceived risk of COVID-19 increased. An error bars graph (small bar = more reliable; larger bar = less reliable) showed that a medium level of perceived risk of COVID-19 was more likely to lead to reliable outcomes in terms of telemedicine use across all the domains compared to both low and high levels of perceived risk.

## 4. Discussion

Telemedicine can mitigate infection transmission and optimise healthcare during pandemics, thereby aiding resource efficiency and the wellbeing of clinicians, patients, and their family members [41]. This study explored the association between the perceived risk of COVID-19 and telemedicine use in hospital settings in Bangladesh.

A total of 550 patients participated in this study—most were male, aged 18 to 25 years, single, from a high-income family, and highly educated. This reflects the literature suggesting that low education, poverty, older age, and being female are discriminatory barriers to telemedicine use [42]. Telephone calls were the most common mode of telemedicine. This might be because telehealth in Bangladesh is supported by limited infrastructure, including reliable internet connectivity [43].

The perceived risk of COVID-19 was moderately high—this suggests that risk perception aided preventative health behaviours, including telemedicine, in response to the COVID-19 pandemic [44]. Consistent with the existing literature [45,46], higher education and income were associated with higher COVID-19 perceived risk. This suggests that education and income aid a recognition of the risk of infection [47] and might increase the likelihood of protective behaviours, such as telemedicine.

Although the participants were mainly satisfied with telemedicine, finding it beneficial and accessible, many were concerned about privacy, care personnel expertise, and its usability. Similarly, other studies reported that telemedicine saves travel time, reduces wait times, and improves the quality of care [48,49]. However, the use of telemedicine is hindered by concerns about: the absence of a physical examination; confidentiality; healthcare professionals’ competencies; and comfort [13,14]. Perceptions of telemedicine varied across demographic variables. For instance, male participants were more likely to be satisfied with telemedicine, finding it accessible and usable; furthermore, they were less likely to be concerned about their healthcare professional’s expertise in delivering telemedicine. While married participants were less likely to find telemedicine accessible, they were less concerned about privacy and discomfort. Yet those who were educated and had a high family income were more likely to be satisfied with telemedicine, finding it beneficial and useful; however, they were concerned about privacy and discomfort. Older participants were concerned about their healthcare professional’s expertise in delivering telemedicine. These findings demonstrate how demographic variables influence perceptions about telemedicine. As such, tailoring telemedicine approaches to accommodate these demographic differences might improve patient engagement with telemedicine.

Although participant perceptions of telemedicine were not dependent on their health issues, these issues did influence some of their perceptions. For instance, while those with arthritis reported positive views, those with pain did not. This warrants further studies because pain is one of the main symptoms of arthritis. Similarly, while telemedicine was accessible to those with lung disease, it was less so for those with infectious disease. Participants with arthritis, hyperlipidaemia, immune diseases, and pain were less likely to raise privacy and discomfort concerns—yet those with mental health or sexual health issues were; this might be partly explained by the social discrimination and stigma often associated with mental health and sexual health issues [50,51]. Only those with eye disease were concerned about their healthcare professional’s expertise in delivering telemedicine.

Collectively, the aforesaid findings have considerable implications for the future of telemedicine. They suggest there might be value in: tailoring telemedicine to accommodate the needs and preferences of patients with particular health issues; ensuring robust confidentiality regulations when addressing health issues associated with social discrimination and stigma [52,53,54,55]; and managing patient expectations of telemedicine.

The perceived risk of COVID-19 strongly predicted all telemedicine domains including perceived benefit, accessibility, and satisfaction, privacy and discomfort, care personnel expertise, and usability after controlling the effect of demographic variables (see Table 6). As such, risk perception was associated with telemedicine use among the participants. This is because the perception of risk encouraged telemedicine use, helping participants to protect themselves against COVID-19 [56]. Specifically, the finding suggests that the perception of risk can be a reliable tool to promote telemedicine use to avoid COVID-19 transmission [44] and control future pandemics. Furthermore, the finding showed that, when participants experienced a greater perceived risk of COVID-19, they were less likely to be concerned about privacy and the expertise of their healthcare professional in delivering telemedicine. These findings might be explained by two reasons, which require further study. First, a participant’s concerns about privacy and their healthcare professional’s expertise in delivering telemedicine might be overstated due to misinformation or limited information about telemedicine and their healthcare professional’s expertise. Second, a high perceived risk of COVID-19 might diminish concerns about safety. Importantly, the findings revealed that a medium level of perceived risk of COVID-19 was associated with telemedicine use, compared to low and high levels of perceived risk. This has important implications for policymakers, suggesting that telemedicine use might be encouraged by addressing low and high perceptions of risk.

## 5. Limitations

Despite the value of the findings reported in this article, two limitations warrant mention. First, given the recruitment process and the convenience sample, there are no claims that the findings can be generalised further afield, within or beyond Bangladesh. Second, given the cross-sectional study design, the findings are likely to have a limited lifespan and causal relationships could not be determined. Given these limitations, there is considerable opportunity for further research with different samples and study designs—this might include co-designing interventions with different patient groups to promote telemedicine use and testing these.

## 6. Conclusions

Although the participants in this study largely found telemedicine to be acceptable, their perceptions were shaped by demographic variables and health issues. The perceived risk of COVID-19 was a strong predictor of telemedicine use—this suggests that risk perception can be used as a reliable tool to promote telemedicine as a risk reduction strategy for COVID-19 and other similar outbreaks in the future. Interestingly, higher COVID-19 perceived risk triggered fewer concerns about privacy and care personnel’s telemedicine expertise, which warrants further studies. These findings provide a strong platform for future research to bolster telemedicine use as a risk reduction strategy for COVID-19 and future pandemics.

## Figures and Tables

**Table 1:** Participants’ demographic variables, health data, and telemedicine modes (*n* = 550).

Demographic Variables	Frequency		Health Data	Frequency	
	*n*	%		*n*	%
Gender			Health issue when telemedicine used		
Male	365	66.4	Heart disease	104	18.9
Female	185	33.6	Arthritis	20	3.6
Age * (years)			Stomach/bowel disease	77	14.0
18–25	255	46.4	Hyperlipidaemia	16	2.9
26–40	228	41.5	Immune system disease	22	4.0
≥41	67	12.2	Sexual or mental health issue	60	10.9
Marital Status			Eye disease	13	2.4
Single	320	58.2	Skin disease	85	15.5
Married	205	37.3	Diabetes	14	2.5
Divorced/Widowed/Other	25	4.5	Pain	28	5.1
Education Level			Lung disease	20	3.6
No Education	25	4.5	Infectious disease	19	3.5
Primary School	41	7.5	Other disease	72	13.1
Secondary School	76	13.8	Telemedicine method		
University/College	408	74.2	Telephone	305	55.4
Employment Status			Live video chat	104	18.9
Full-time	205	37.3	Telephone message	68	12.4
Part-time/Casual ****	72	13.1	Forwarding medical documents to specialist	73	13.3
Unemployed ***	273	49.6			
Family Income Status **					
Low Income	65	11.8			
Middle Income	222	40.4			
High Income	263	47.8			

* Age: mean = 30.2 (±12.3) years, minimum = 18, maximum = 85. ** Low income = less than BDT 5000 per month, middle income = BDT 5000–20,000 per month, high income = more than BDT 20,000 per month. BDT is the currency for Bangladesh. *** Unemployed participants did not have any job or employment at the time of survey. **** Casual employment referred to a temporary and flexible job without any ongoing work commitment or requirements beyond completing a job.

**Table 2:** Perceived risk of COVID-19 (*n* = 550).

Item	Frequency					
	*n*	%	*n*	%	*n*	%
What is your gut feeling about how likely you are to get infected with COVID-19?	Very/Extremely Unlikely		Somewhat Likely		Very/Extremely Likely	
	96	17.5	180	32.7	274	49.8
Picturing myself getting COVID-19 is something I find	Hard/very hard to do		Easy to do		Extremely/very easy to do	
	62	11.3	141	25.6	347	63.1
I am sure I will NOT get infected with COVID-19	Agree/strongly agree		Somewhat agree		Disagree/strongly disagree	
	98	17.8	78	14.2	374	68.0
I feel I am unlikely to get infected with COVID-19	Agree/strongly agree		Somewhat agree		Disagree/strongly disagree	
	143	26.0	108	19.6	299	54.4
I feel vulnerable to COVID-19 infection	Strongly disagree/disagree		Somewhat agree		Agree/strongly agree	
	191	34.7	155	28.2	204	37.1
I think my chances of getting infected with COVID-19 are	Zero/small		Moderate		Large/very large	
	78	14.2	157	28.5	315	57.3
Comparison of COVID-19 perceived risk across significant demographic variables (*n* = 550)						
COVID-19 Perceived Risk	Mean Difference		±SD		95% Confidence Interval of the Difference	
Primary school versus no education	3.5		±0.9 ***		5.6–1.6	
Secondary school versus no education	4.1		±0.8 ***		5.3–1.6	
University/college versus no education	4.8		±0.7 ***		5.4–2.1	
	ANOVA, *f* = 9.1 ***, *df* = 3					
Middle income versus low income	1.1		±0.5 *		2.2–0.9	
High income versus low income	1.2		±0.6 *		2.1–0.3	
	ANOVA, *f* = 2.8 *, *df* = 2					

M = 21.5 (±SD = 3.6), minimum = 8, maximum = 30, * *p* < 0.05, ** *p* < 0.01, and *** *p* < 0.001.

**Table 3:** Participants’ perceptions about different domains of telemedicine use (*n* = 550).

Item	Frequency							
	Moderate/Strongly Agree		Mildly Agree		Mildly Disagree		Moderately/Strongly Disagree	
	*n*	%	*n*	%	*n*	%	*n*	%
Perceived benefit	M = 22.3 (±SD = 4.5), Minimum = 6, Maximum = 30							
The telemedicine or telecare has allowed me to be less concerned about my health and/or social care	198	36.0	168	30.5	99	18.0	85	15.5
The telemedicine or telecare has made me more actively involved in my health	291	53.0	183	33.2	46	8.3	30	5.5
The telemedicine or telecare allows the people looking after me, to better monitor me and my condition	276	50.2	177	32.2	54	9.8	43	7.8
The telemedicine or telecare can be/should be recommended to people in a similar condition to mine	343	62.4	132	24.0	37	6.7	38	6.9
The telemedicine or telecare can certainly be a good addition to my regular health or social care	291	53.0	155	28.1	60	10.9	44	8.0
Accessibility	M = 18.6 (±SD = 3.6), Minimum = 8, Maximum = 24							
The telemedicine or telecare I received has saved me time in that I did not have to visit my GP clinic or other health/social care professional as often	309	56.1	166	30.2	46	8.4	29	5.3
The telemedicine or telecare I received has increased my access to care (health and/or social care professionals)	307	55.8	162	29.5	61	11.1	20	3.6
The telemedicine or telecare I received has helped me to improve my health	296	53.8	173	31.5	60	10.9	21	3.8
The telemedicine or telecare has made it easier to get in touch with health and social care professionals	305	55.4	167	30.4	45	8.2	33	6.0
Privacy and discomfort	M = 14.4 (±SD = 4.5), Minimum = 4, Maximum = 24							
The telemedicine or telecare I received has interfered with my everyday routine	188	34.2	162	29.5	67	12.2	133	24.1
The telemedicine or telecare I received has invaded my privacy	168	30.5	148	26.9	76	13.8	158	28.7
The telemedicine or telecare has made me feel uncomfortable, (e.g., physically or emotionally)	140	25.5	122	22.2	105	19.1	183	33.2
The telemedicine or telecare makes me worried about the confidentiality of the private information being exchanged through it	186	33.8	161	29.3	96	17.5	107	19.4
Care personnel concerns	M = 12.5 (±SD = 2.8), Minimum = 3, Maximum = 18							
I am concerned about the level of expertise of the individuals who monitor my status via the telemedicine or telecare	241	44.0	181	32.9	64	11.6	63	11.5
The telemedicine or telecare interferes with the continuity of the care I receive (i.e., I do not see the same care professional each time)	231	42.0	177	32.2	67	12.2	75	13.6
I am concerned that the person who monitors my status, through the telemedicine or telecare, does not know my personal health/social care history	238	42.5	177	32.2	48	8.7	91	16.6
Usability	M = 10.5 (±SD = 2.7), Minimum = 3, Maximum = 18							
The telemedicine or telecare can be a replacement for my regular health or social care	215	39.1	159	28.8	88	16.0	89	16.1
The telemedicine or telecare is not as suitable as regular face to face consultations with the people looking after me	228	41.5	184	33.5	89	16.1	49	8.9
The telemedicine or telecare has allowed me to be less concerned about my health status	177	32.1	138	25.1	117	21.3	118	21.5
Satisfaction	M = 14.0 (±SD = 3.1), Minimum = 3, Maximum = 18							
The telemedicine or telecare has been explained to me sufficiently	324	58.9	127	23.1	54	9.8	45	8.2
The telemedicine or telecare can be trusted to work appropriately	338	61.5	129	23.5	48	8.6	35	6.4
I am satisfied with the telemedicine or telecare I received	336	61.1	136	24.7	39	7.1	39	7.1

**Table 4:** Comparison of telemedicine domains across significant demographic variables (*n* = 550).

Telemedicine Use	Mean Difference	±SD	95% Confidence Interval of the Difference
Perceived benefit			
Primary school versus no education	5.1	±1.1 ***	7.6–2.5
Secondary school versus no education	4.0	±1.0 ***	6.3–1.6
University/college versus no education	4.5	±0.9 ***	6.5–2.3
	ANOVA, *f* = 8.5 **, *df* = 3		
Middle family income versus low income	1.1	±0.5 *	2.2–0.9
High family income versus low income	1.2	±0.6 *	2.1–0.3
	ANOVA, *f* = 2.8 *, *df* = 2		
Accessibility			
Male versus female	1.6	±0.3 *	0.1–1.2
	*t*-test, *t* = 1.9 *, *df* = 548		
Married versus single	−0.7	±0.3 *	−1.4–−0.1
	ANOVA, *f* = 2.7 *, *df* = 2		
Primary school versus no education	3.2	±0.9 **	5.2–1.1
Secondary school versus no education	2.2	±1.0 *	4.1–0.3
University/college versus no education	2.4	±0.9 **	4.1–0.7
	ANOVA, *f* = 4.3 **, *df* = 3		
Privacy and discomfort			
Married versus single	−1.0	±0.4 *	−1.8–−1.0
	ANOVA, *f* = 3.2 *, *df* = 2		
University/college versus no education	2.5	±0.9 *	0.4–4.5
	ANOVA, *f* = 3.9 **, *df* = 3		
Part-time/casual versus full-time	1.9	±0.6 **	0.5–3.2
	ANOVA, *f* = 4.7 **, *df* = 2		
High family income versus low income	1.3	±0.6 *	−0.9–2.5
	ANOVA, *f* = 6.2 **, *df* = 2		
Care personnel concerns			
Male versus female	−1.5	±0.2 *	−1.1–−0.2
	*t*-test, *t* = −2.1 *, *df* = 548		
26–40 years of age versus 18–25 years of age	0.7	±0.2 *	−0.8–1.2
≥41 years of age versus 18–25 years of age	1.1	±0.6 *	−0.2–1.9
	ANOVA, *f* = 5.3 **, *df* = 2		
Usability			
Male versus female	0.5	±0.2 *	0.02–0.9
	*t*-test, *t* = −2.3 *, *df* = 548		
Primary school versus no education	2.8	±0.7 **	4.3–1.2
Secondary school versus no education	2.5	±0.6 **	3.9–1.1
University/college versus no education	2.6	±0.5 **	3.8–1.3
	ANOVA, *f* = 7.8 **, *df* = 3		
High family income versus low income	1.2	±0.3 **	1.9–0.4
	ANOVA, *f* = 8.7 **, *df* = 2		
Satisfaction			
Male versus female	0.9	±0.3 **	0.3–1.4
	*t*-test, *t* = −3.1 **, *df* = 548		
Primary school versus no education	3.3	±0.7 **	−5.1–−1.5
University/college versus no education	1.8	±0.5 *	−3.3–−0.4
	ANOVA, *f* = 6.8 **, *df* = 3		
Middle family income versus low income	1.1	±0.4 *	−0.02–1.9
High family income versus low income	0.9	±0.4 *	0.6–1.2
	ANOVA, *f* = 3.3 *, *df* = 2		

* *p* < 0.05, ** *p* < 0.01, and *** *p* < 0.001.

**Table 5:** Comparison of different domains of telemedicine use across different types of diseases (*n* = 550).

Disease	Perceived Benefit	Accessibility	Privacy and Discomfort	Care Personnel Concerns	Usability	Satisfaction
	MD ^#^ (±SD ^##^) [95% CI ^###^]	MD ^#^ (±SD ^##^) [95% CI ^###^]	MD ^#^ (±SD ^##^) [95% CI ^###^]	MD ^#^ (±SD ^##^) [95% CI ^###^]	MD ^#^ (±SD ^##^) [95% CI ^###^]	MD ^#^ (±SD ^##^) [95% CI ^###^]
Heart disease versus other diseases	Not significant	Not significant	Not significant	Not significant	0.7 * (±0.3) [0.1–1.2]	Not significant
Arthritis versus other diseases	2.4 ** (±0.6) [1.1–3.6]	Not significant	−2.4 ** (±0.5) [−3.5–−1.3]	Not significant	Not significant	1.3 * (±0.5) [0.3–2.3]
Stomach/bowel diseases versus other diseases	Not significant	Not significant	Not significant	Not significant	Not significant	Not significant
Hyperlipidaemia versus other diseases	Not significant	Not significant	−2.9 ** (±0.5) [−3.9–−1.7]	Not significant	Not significant	Not significant
Immune diseases versus other diseases	Not significant	Not significant	−2.5 ** (±0.6) [−3.8–−1.2]	Not significant	Not significant	Not significant
Sexual/mental health versus other diseases	Not significant	Not significant	3.0 ** (±0.7) [1.6–4.3]	Not significant	−0.8 * (±0.4) [−1.6–−0.1]	Not significant
Skin diseases versus other diseases	Not significant	Not significant	Not significant	Not significant	Not significant	Not significant
Diabetes versus other diseases	Not significant	Not significant	Not significant	Not significant	Not significant	Not significant
Pain versus other diseases	−1.8 * (±0.8) [−3.5–−0.1]	Not significant	−1.5 * (±0.6) [−2.9–−0.1]	Not significant	Not significant	Not significant
Lung diseases versus other diseases	Not significant	−1.7 * (±0.5) [−2.8–−0.6]	Not significant	Not significant	Not significant	Not significant
Eye diseases versus other diseases	Not significant	Not significant	Not significant	1.5 * (±0.6) [2.8–0.3]	Not significant	Not significant
Infectious diseases versus other diseases	Not significant	2.1 ** (±0.7) [0.5–3.5]	1.7 * (±0.7) [0.3–3.0]	Not significant	Not significant	Not significant

MD ^#^ = mean difference, SD ^##^ = standard deviation, CI ^###^ = confidence interval, * *p* < 0.05, ** *p* < 0.01 and *** *p* < 0.001.

**Table 6:** Summary of multiple regression analyses for COVID-19 perceived risk predicting different domains of telemedicine use while controlling demographic variables (*n* = 550).

Criterion	Perceived Benefit			Accessibility			Privacy and Discomfort		
Predictors	B (β) ^###^	*t ^####^* Value	UV (%) ^#^	B (β) ^###^	*t ^####^* Value	UV (%) ^#^	B (β) ^###^	*t ^####^* Value	UV (%) ^#^
COVID-19 perceived risk	0.6 (0.5)	11.4	19.4 **	0.6 (0.5)	14.0	26.6 **	−0.5 (−0.4)	−10.4	−16.8 **
≥41 years of age versus 18–25 years of age							2.8 (0.2)	3.6	2.4 **
Married versus single							1.6 (0.2)	3.7	−2.5 **
Primary school versus no education	2.7 (0.2)	2.6	1.3 *						
Secondary school versus no education	3.1 (0.3)	2.9	1.6 **						
University/college versus no education	3.6 (0.2)	3.4	2.1 **						
Part-time/casual versus full-time							1.5 (0.1)	2.6	1.3 *
	(*R*^2^ = 25.7%, *df* = 13, *f* = 14.3 **)			(*R*^2^ = 30.1%, *df* = 13, *f* = 17.8 **)			(*R*^2^ = 24.4%, *df* = 13, *f* = 13.3 **)		
	Care Personnel Concerns			Usability			Satisfaction		
COVID-19 perceived risk	−0.3 (−0.4)	−8.9	−13.0 **	0.3 (0.4)	9.9	15.5 ***	0.4 (0.4)	11.4	19.6 **
26–40 years of age versus 18–25 years of age	0.8 (0.2)	2.9	1.6 *						
≥41 years of age versus 18–25 years of age	1.5 (0.2)	2.9	1.5 *						
Middle family income versus low income							1.5 (0.2)	3.4	2.2 **
	(*R*^2^ = 16.5%, *df* = 13, *f* = 8.1 **)			(*R*^2^ = 22.2%, *df* = 13, *f* = 11.2 **)			*R*^2^ = 25.5%, *df* = 13, *f* = 14.2 **)		

UV (%) ^#^ = unique variance because of variable (%), * *p* < 0.05, ** *p* < 0.01, *** *p* < 0.001. ^###^ B is the unstandardized beta, representing the slope of the line between the predictor and the dependent variables. It shows for every one unit increase in the predictor, the dependent variable will increase by how much. β is the standardized beta ranging from 0 to 1 or 0 to −1, depending on the direction of the relationship. The closer the value is to 1 or −1, the stronger the relationship. ^####^ *t* is the *t*-test statistic value, which is used to calculate the *p*-value for each variable.

## Data Availability

The data presented in this study are available on request from the corresponding author. The data are not publicly available due to privacy restrictions.

## Supplementary Materials

The following supporting information can be downloaded at: https://www.mdpi.com/article/10.3390/ijerph20043061/s1, File S1: Study advertisement flyer and survey.
