# Supplementary material for: Telemedicine Use and the Perceived Risk of COVID-19: Patient Experience
Source: Int J Environ Res Public Health. 2023 Feb 9;20(4):3061. doi: 10.3390/ijerph20043061 (PMC9960459; doi:10.3390/ijerph20043061)
Supplement: Supplementary file 1 [file ijerph-20-03061-s001.zip › ijerph-2142885-supplementary.pdf]

Study advertisement flyer

Have you used telemedicine during the COVID 19 pandemic!
Volunteers Needed for Research study on Telemedicine during the COVID 19 pandemic

We are looking for patients who have used telemedicine at least once during the COVID 19 pandemic.

This study titled "Patient experience with telemedicine: A risk reduction approach to COVID-19 management" aims to understand the barriers and facilitators of using telemedicine as risk reduction strategy among clinicians and patients during COVID 19 in hospital settings in Bangladesh.

<u>Patients will be eligible if they</u>:

- Are aged over 18 years
- Have used telemedicine in hospital settings during COVID 19 outbreak at least once
- Do not have any cognitive impairment (e.g. Alzheimer)

<u>Participants will involve:</u>

- Anonymous online and paper surveys. They can choose whichever you prefer.

<u>If you are unsure if you meet the requirements, call or email a member of the study team:</u>

Name: XXX
Email: XX
Phone: XXX

Telemedicine and COVID-19 management survey

Welcome to Patient experience with telemedicine: A risk reduction approach to COVID-19 management! Through this study, we will be able to improve telemedicine use in hospital settings in Bangladesh. Your responses are strictly confidential and would only be used for research purposes.

Demographics Information
Gender: 1Male ☐ 2Female ☐ 3 Prefer not to say ☐
Age ……. (Year)

Marital status
☐ 1Single
☐ 2Married
☐ 3Divorced
☐ 4Widowed
☐ 5Other (please specify…………………………………………………….)

Education level
☐ 1No education
☐ 2Primary incomplete
☐ 3Primary complete
☐ 4Secondary incomplete
☐ 5Secondary complete
☐ 6University or college qualification

Employment status: (tick one box)
1Full time ☐ 2Part time ☐ 3Casual ☐ 4Unemployed ☐

Family Income status
☐ 1 Less than 5000tk per month
☐ 2 5000tk to 10000tk per month
☐ 3 10000tk to 20000tk per month
☐ 4 More then 20000tk per months

Type of disease (s) you had when you used telemedicine or telecare

☐ Blood Pressure	☐ Arthritis	☐ Stomach or Bowel Problems
☐ High Cholesterol	☐ Thyroid Disease	☐ Sexual Problems
☐ High Triglycerides	☐ Depression	☐ Skin Problems
☐ Diabetes	☐ Kidney Disease	☐ Pain or Fatigue Syndromes
☐ Heart Disease	☐ Lung disease	☐ Osteoporosis
☐ Stroke	☐ Eye Disease	☐ Cancer

☐ Other (please specify) ……………………………………………………………….

What type of telemedicine method did your doctor use?
Phone ☐ Live video chat ☐ Phone message ☐ Forwarding your medical documents to your specialist ☐ Other ☐ (please specify…………………………………………………………………………)

Below are some statements that people sometimes make when they talk about their risk of COVID 19. Please indicate how much you agree or disagree with each statement as it applies to you personally by placing a cross in the appropriate box. There are no right or wrong answers, just what is true for you.

Please circle the appropriate response. (Cross one answer for each line)					
1. What is your gut feeling about how likely you are to get infected with COVID-19?	Extremely unlikely	Very unlikely	Somewhat likely	Very likely	Extremely likely
2. Picturing myself getting COVID-19 is something I find	Very hard to do	Hard to do	Easy to do	Very easy to do	Extremely easy to do
3. I am sure I will NOT get infected with COVID-19	Strongly disagree	Somewhat disagree	Somewhat agree	Agree	Strongly agree
4. I feel I am unlikely to get infected with COVID-19	Strongly disagree	Disagree	Somewhat agree	Agree	Strongly agree
5. I feel vulnerable to COVID-19 infection	Strongly disagree	Disagree	Somewhat agree	Agree	Strongly agree
6. I think my chances of getting infected with COVID-19 are	Zero	Small	Moderate	Large	Very Large

Below is a list of statements referring to the telemedicine or telecare you have received to support your care. Please indicate the degree to which you agree with each statement by crossing the corresponding box.

Please circle the appropriate response. (Cross one answer for each line)	Strongly agree	Moderately agree	Mildly agree	Mildly disagre e	Moderately disagree	Strongly Disagree
1. The telemedicine or telecare I received has saved me time in that I did not have to visit my GP clinic or other health/social care professional as often.	1	2	3	4	5	6
2. The telemedicine or telecare I received has interfered with my everyday routine.	1	2	3	4	5	6
3. The telemedicine or telecare I received has increased my access to care (health and/or social care professionals).	1	2	3	4	5	6
4. The telemedicine or telecare I received has helped me to improve my health.	1	2	3	4	5	6
5. The telemedicine or telecare I received has invaded my privacy.	1	2	3	4	5	6
6. The telemedicine or telecare has been explained to me sufficiently.	1	2	3	4	5	6
7. The telemedicine or telecare can be trusted to work appropriately.	1	2	3	4	5	6
8. The telemedicine or telecare has made me feel uncomfortable, e.g. physically or emotionally.	1	2	3	4	5	6
9. I am concerned about the level of expertise of the individuals who monitor my status via the telemedicine or telecare.	1	2	3	4	5	6
10. The telemedicine or telecare has allowed me to be less concerned about my health and/or social care.	1	2	3	4	5	6
11. The telemedicine or telecare has made me more actively involved in my health.	1	2	3	4	5	6

12. The telemedicine or telecare makes me worried about the confidentiality of the private information being exchanged through it.	1	2	3	4	5	6
13. The telemedicine or telecare allows the people looking after me, to better monitor me and my condition.	1	2	3	4	5	6
14. I am satisfied with the telemedicine or telecare I received.	1	2	3	4	5	6
15. The telemedicine or telecare can be/should be recommended to people in a similar condition to mine.	1	2	3	4	5	6
16. The telemedicine or telecare can be a replacement for my regular health or social care.	1	2	3	4	5	6
17. The telemedicine or telecare can certainly be a good addition to my regular health or social care.	1	2	3	4	5	6
18. The telemedicine or telecare is not as suitable as regular face to face consultations with the people looking after me.	1	2	3	4	5	6
19. The telemedicine or telecare has made it easier to get in touch with health and social care professionals.	1	2	3	4	5	6
20. The telemedicine or telecare interferes with the continuity of the care I receive (i.e. I do not see the same care professional each time).	1	2	3	4	5	6
21. I am concerned that the person who monitors my status, through the telemedicine or telecare, does not know my personal health/social care history.	1	2	3	4	5	6
22. The telemedicine or telecare has allowed me to be less concerned about my health status.	1	2	3	4	5	6
